# High serum alkaline phosphatase cooperating with MMP-9 predicts metastasis and poor prognosis in patients with primary osteosarcoma in Southern China

**DOI:** 10.1186/1477-7819-10-37

**Published:** 2012-02-15

**Authors:** Ju Han, Bicheng Yong, Canqiao Luo, Pingxian Tan, Tingsheng Peng, Jingnan Shen

**Affiliations:** 1 1st Affiliated Hospital of Sun Yat-sen University, Pathological Department, Guangzhou, P R China; 2 1st Affiliated Hospital of Sun Yat-sen University, Musculoskeletal Oncology Department, Guangzhou, P R China

**Keywords:** ALP, MMP-9, metastasis, prognosis, osteosarcoma

## Abstract

**Background:**

Osteosarcoma is a malignant tumor with high ability to form invasion and metastasis. Identifying prognostic factor in osteosarcoma is helpful to select those patients for more aggressive management. Our study evaluated serum alkaline phosphatase (ALP) cooperating with matrix metalloproteinase-9 (MMP-9) as an important prognostic predictor for local recurrence and distant metastasis of osteosarcoma.

**Methods:**

177 cases were included from the osteosarcoma patients treated at 1st Affiliated Hospital of Sun Yat-sen University (1999-2008). Pre-chemotherapy serum ALP (pre-ALP) were studied and correlated with tumor recurrence, lung metastasis and patient survival. MMP-9 protein in tumor tissues was detected by immunohistochemistry and correlated with pre-ALP level.

**Results:**

Pre-ALP were partitioned into normal, high, and very high groups, in each group the incidence of metastases was 12.2%, 21.2% and 34.6%, respectively (p = 0.007). In the three groups the mean disease-free survival (DFS) was 57 ± 3.15, 28 ± 3.57 and 14 ± 3.35 months, respectively (p < 0.001); overall survival (OS) was 92 ± 26.89, 39 ± 8.61 and 17 ± 5.07 months, respectively (p < 0.001). By multivariate analysis, elevated serum pre-ALP were associated with shorter DFS (p = 0.018) and OS (p = 0.031). If elevated ALP levels decreased after clinical treatment, the incidence of lung metastasis rate decreased (p = 0.028); DFS and OS were both prolonged (p < 0.001). Pre-ALP was also positively correlated with MMP-9 expression (p = 0.015) in tumor tissue.

**Conclusions:**

Pre-ALP was an independent prognostic factor for the survival of osteosarcoma patients in south China, and correlated with MMP-9 expression and lung metastasis. ALP can also serve as a prognostic marker for treatment, and merit large-scale validation studies.

## Background

Osteosarcoma is a malignant bone tumor that typically occurs in children, adolescents and young adults. Incorporation of chemotherapy into initial treatment significantly increased the cure rate. However, about 40% of patients died from lung metastases [1]; it will be important to develop biomarkers that can inform therapy while offering prognostic insight, especially in identifying poor prognosis patients who should be offered more aggressive therapy at an early time point in the clinical continuum [2-4].

Serum alkaline phosphatase (ALP) can be easily demonstrated in osteosarcoma patients, which may be predictive of survival [5-8]. However this remains controversial [9]. Alkaline phosphatase has been addressed as a prognostic factor by several authors with inconclusive results. Only a few previous studies have studied the ALP levels in osteosarcoma patients after chemotherapy and surgery [10] or cases of local recurrence or lung metastasis after surgery [11], or have considered MMP-9 expression in these contexts [12-14]. Accordingly, we examined whether or not serum ALP was predictive of lung metastasis, local recurrence and survival after surgery of osteosarcoma patients in south China. As known, MMP-9 expression and function is involved during lung metastasis of tumor, so we concerned whether serum ALP was connected with MMP-9 expression.

## Methods

### Patients and methods

**Figure 1:**
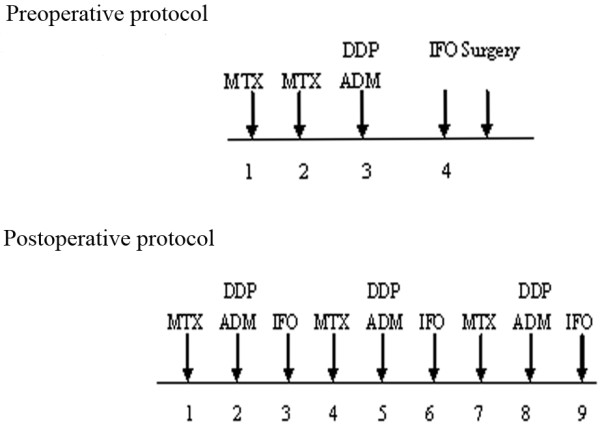
**The chemotherapeutic agents and the treatment protocol of 177 osteosarcoma patients**.

The study population consisted of 177 patients who presented at 1st Affiliated Hospital of Sun Yat-sen University with primary non-metastatic osteosarcoma from 1999 to 2008. Methotrexate(MTX), cisplatin (DDP), doxorubicin (ADM), ifosfamide (IFO) were using during chemotherapy. All patients received neo-chemotherapy followed by surgical resection and post-operative chemotherapy (Figure 1); "standard" chemotherapy consisting of 4 neo-chemotherapy courses and 8 or more post-operative chemotherapy courses.

Fasting morning blood samples were collected at diagnosis, analyzed for pre-chemotherapy serum ALP (pre-ALP). Because growth influences ALP expression [15], 150 U/L was considered as the upper normal serum ALP limit in patients less than 18 years, and 110 U/L in those 18 years or older. Pre-ALP values were divided into three categories: normal (below the upper normal limit), high (elevated to less than twice the upper limit), and very high (elevated more than twice the upper limit).

### MMP-9 Immunohistochemistry

Tumor biopsy samples from these patients (n = 97) were cut into 4 μm thick serial tissue sections. Sections were deparaffined in xylene and rehydrated in grade alcohols. The sections were then treated with 3% hydrogen peroxide for 30 min to quench the endogenous peroxidase activity. Antigenic retrieval was performed by submerging in citric acid (pH = 6.0) and microwaving. The slides were then allowed to cool at room temperature. The sections were then incubated with primary antibodies for MMP-9 (mouse monoclonal, MAB-0245, maixin Biological Technology, Fuzhou) overnight at 4°C; phosphate-buffered saline (PBS) was used as negative control. After washing with PBS at room temperature, goat anti-mouse antibody conjugated to a horseradish peroxidase decorated dextran polymer backbone (Envision, Dako, Denmark), was applied for 30 min at 37°C. Staining was carried out with 3, 3'-diaminobenzidine tetrahydrochloride. Mayer's hematoxylin was applied as a counter stain.

Each section was evaluated by three independent pathologists without knowledge of the clinical case features. We selected 100 cells from each of 5 fields and evaluated for MMP-9 positivity. Staining results were scored semiquantitatively based on the combined percentage (five-tiered algorithm [0: 0% positive cells; 1: < 25% positive cells; 2: 25%-50% positive cells; 3: 56%-75% positive cells; 4: > 75% positive cells]) and intensity of cytoplasmic staining (four-tiered system [0: negative; 1: weak; 2: moderate; 3: strong]), then tabulated as an MMP-9 expression index (percentage positive) × (intensity)[16,17]. The indexes separately from all the three pathologists were averaged as the final MMP-9 expression index.

### Statistical analysis

Disease-free survival (DFS) was calculated as time from the date of diagnosis to the date of first local recurrence or metastatic failure after surgery or to the date of last follow-up in patients without recurrence or metastasis. Overall survival (OS) was calculated as the time from the date of diagnosis to the date of death or the date of last follow-up if the patient was still alive. Survival rate was calculated using the Kaplan-Meier method. Univariate and multivariate survival analyses were performed to test the association of clinicopathological features with DFS and OS, incorporating log-rank testing and Cox proportional hazard regression models. Correlations between pre-ALP and clinicopathological features were examined by chi-square or ANOVA testing. Pearson analyses were used to test correlations between pre-ALP and MMP-9 expression in osteosarcoma tissues. Statistical analyses were conducted using SPSS 16.0 (SPSS, Inc., Chicago, IL, USA) with a 2-sided significance level of P < 0.05.

## Results

### Patient clinical characteristics

Primary osteosarcoma patients with no metastasis at diagnosis (n = 177) were analyzed; 117 male and 60 female patients. The median age was 18.73 years (range: 6-56 years); 72 patients were older than 18 years of age. Tumors were located in the femur (93/177), tibia (52/177), fibula (13/177), humerus (12/177), radius (1/177) and other sites (6/177). Histological subtypes included 109 osteoblastic, 25 chondroblastic, 16 fibroblastic, 11 dilated blood vessel and 16 miscellaneous osteosarcoma subtypes. Limb sparing surgery was performed in 92 patients whereas 85 underwent amputation.

The median follow-up was 87 months (range: 8-144 months). During follow-up 40 (22.6%) and 14 (7.9%) patients had lung metastases and local recurrence, respectively. The lung metastasis and/or local recurrence were diagnosed by both imaging and pathology. The median OS and DFS of patients were 28 months (95% confidence interval [CI], 32.84-41.95 months) and 24 months (95% CI, 27.79-36.84 months), respectively; 96 patients died of tumor-related causes during the study.

### Correlation between pre-ALP and clinicopathologic characteristics

As shown in Table 1, of the 40 patients who developed lung metastasis, 6 cases were in the normal pre-ALP, 16 cases were in the high pre-ALP, and 18 cases were in the very high pre-ALP groups; while the rates of lung metastasis in the three groups were 12.2%, 21.2% and 34.6%, respectively (p = 0.007). It showed that pre-ALP levels were positively correlated with lung metastasis. Moreover, pre-ALP levels were inversely related to tumor size and courses of post-operative chemotherapy. Pre-ALP levels were significantly elevated in osteoblastic subtype than in other subtypes, also in femur sites than in other sites (p < 0.01, respectively). No correlation between pre-ALP levels and local recurrence was demonstrable.

**Table 1:** Correlation between pre-ALP and clinicopathologic characteristics

Clinicopathologic characteristics	Very high pre-ALP (*n *= 52)	High pre-ALP (*n *= 76)	Normal pre-ALP (*n *= 49)	*P *value
Gender				
Male	35	50	32	0.831
Female	17	26	17	
				
Primary location				
Humerus	6	2	4	0.005
Radius	0	1	0	
Femur	36	40	17	
Tibia	7	28	17	
Fibula	3	2	8	
Others	0	3	3	
				
Histological type				
Osteoblastic	40	45	24	< 0.01
Chondroblastic	8	12	5	
Fibroblastic	1	9	6	
Dilated blood vessels	3	5	3	
Others	0	5	11	
				
Tumor size				
< 6 cm	14	35	26	0.008
≥ 6 cm	38	41	23	
				
Surgery type				
Amputation	24	35	26	0.268
Limb sparing	28	41	23	
				
Post-operative chemotherapy				
No standard ^a^	40	54	20	< 0.01
Standard ^b^	12	22	29	
				
Lung metastasis				
Yes	18	16	6	0.007
No	34	60	43	
				
Local recurrence				
Yes	4	7	3	0.782
No	48	69	46	
				
Survival status				
Alive	14	35	32	< 0.01
Dead	38	41	17	

ALP alkaline phosphatase

^a ^post-operative chemotherapy cycles < 8

^b ^post-operative chemotherapy cycles ≥ 8

**Figure 2:**
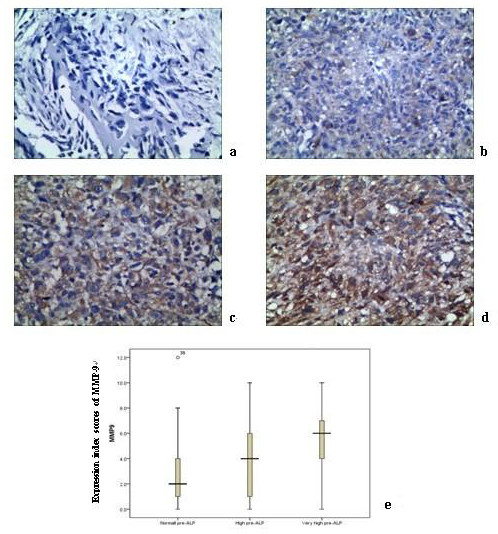
**Immunohistochemical analyses of MMP-9 staining (a: negatives, × 200; b: weak staining, x200; c: moderate staining, × 200; d: strong staining, × 200)**. As can be seen, MMP-9 shows strongest staining in very high pre-ALP group of primary osteosarcoma tissues (e).

The possible correlation between pre-ALP levels and osteosarcoma MMP-9 expression was examined. Immunohistochemistry staining showed that MMP-9 proteins were mainly expressed in tumor cytoplasm and positively correlated with elevated pre-ALP levels (r = 0.250, p = 0.015; Figure 2). The expression of MMP-9 was significantly higher in the very high pre-ALP group compared to the high or normal pre-ALP groups (p = 0.034).

### Pre-ALP is associated with an improved prognosis

**Figure 3:**
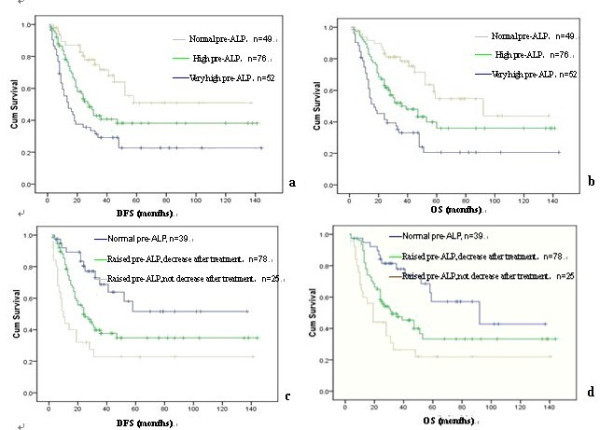
**Influence of serum ALP levels on Disease-free survival (DFS) and Overall survival (OS) Kaplan-Meier curves shows that the patients with high pre-ALP levels have poorer DFS(a) and OS (b); with pre-ALP decreased after clinical treatment have better DFS (c) and OS (d); all of them have significant difference(p < 0.05)**.

**Figure 4:**
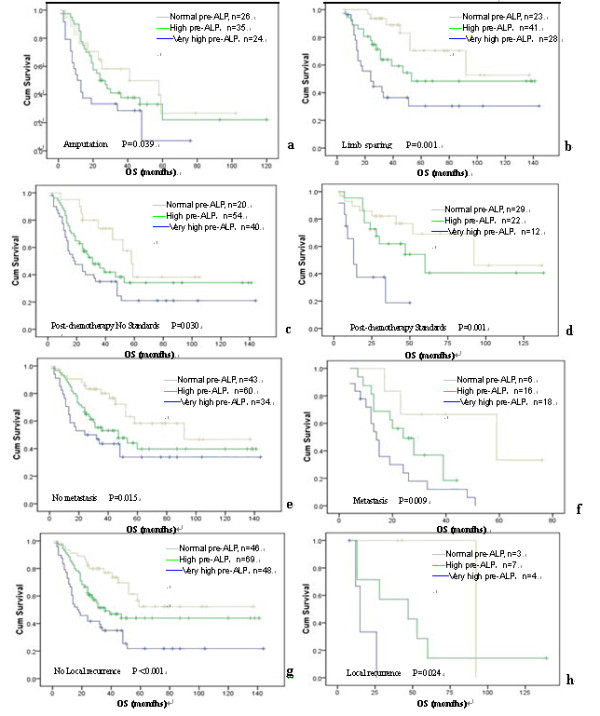
**Overall survival curves were stratified by pre-ALP levels according to surgery style, Post-chemotherapy, metastasis and local recurrence after surgery**. Kaplan-Meier curves shows that the patients with high pre-ALP levels have poorer OS independent of surgery type (a, b), Post-chemotherapy (c, d), metastasis (e, f) and local recurrence after surgery (g, h).

Next in order to elucidate the possible relationship between pre-ALP levels and patient survival, we demonstrated a positive correlation with decreased DFS and OS. The DFS was 57 ± 3.15 months in the normal pre-ALP group, 28 ± 3.57 months in high pre-ALP group, and 14 ± 3.35 months in very high pre-ALP group, respectively (Figure 3a-b; p < 0.001). Furthermore, OS was 92 ± 26.89, 39 ± 8.61 and 17 ± 5.07 months in these same three pre-ALP groups, respectively (p < 0.001). The survival rate was 65.3% (32/49) for patients with normal pre-ALP, while they dramatically dropped to 46.1% (35/76) and 26.9% (14/52) for patients with high pre-ALP and very high pre-ALP, respectively (Table 1; p < 0.01). We also considered whether pre-ALP levels could serve as a prognostic factor regardless of therapeutic approaches. Pre-ALP levels correlated strongly with patient survival even after stratifying for types of surgery, courses of post-chemotherapy, and incidence of lung metastasis or local recurrence (Figure 4a-h).

Given the seeming responsiveness of serum ALP to osteosarcoma clinical treatment [18], we analyzed the ALP level after clinical treatment. If elevated pre-ALP levels decreased after clinical treatment, the incidence of lung metastasis was 28.2% (22/78; p = 0.028) and DFS and OS were 24 ± 5.00 and 32 ± 7.86 months, respectively (p < 0.001). If not, the incidence of lung metastasis was 40.0% (10/25), and DFS and OS were 10 ± 1.87 and 19 ± 3.30 months, respectively (Figure 3c-d). The decrease of pre-ALP was not correlated with local recurrence.

### Analyses of Relative Risk indicate the role of pre-ALP in the prognosis of primary osteosarcoma

We examined DFS and OS using Cox regression hazard analyses to determine whether pre-ALP levels could serve as a clinically useful prognostic assessment factor in osteosarcoma. Univariate Cox regression analyses revealed that elevated pre-ALP levels were associated with inferior survival in primary osteosarcoma patients (Table 2; p < 0.01). The relative risk indicated that osteoblastic osteosarcoma, tumor size ≥ 6 cm, amputation, post-operative chemotherapy not standard, lung metastasis and local recurrence were all worse predictors. After adjusting for potential confounding factors, elevated pre-ALP in osteosarcoma was found to be a poorer survival predictor for DFS and OS in an independent manner (p = 0.018 and 0.031, respectively; Table 3). Using multivariate Cox regression model for clinicalpathologic diagnoses gave rise to the following results: amputation and lung metastasis predicted poorer DFS. Osteoblastic osteosarcoma, tumor size ≥ 6 cm, amputation, and lung metastasis predicted poorer OS. The relative risk showed no obvious differences when including other clinicopathologic features such as gender, primary location and local recurrence in the analyses using the multivariate Cox regression model.

**Table 2:** Univariate Cox regression analysis of potential prognostic factors for 177 osteosarcoma patients

Clinical characteristics	OS		DFS	
	
	Hazard ratio (95% *CI*)	*P*	Hazard ratio (95% *CI*)	*P*
Histological type				
Osteoblastic	4.090(1.491,11.251)	0.006	3.524(1.283,9.677)	0.015
Chondroblastic	2.741(0.882,8.521)	0.081	2.140(0.689,6.648)	0.188
Fibroblastic	1.573(0.422,5.862)	0.500	1.231(0.331,4.588)	0.756
Dilated blood vessels	3.313(0.933,11.767)	0.064	3.249(0.913,11.555)	0.069
Others	1.000		1.000	
				
Tumor size				
< 6 cm	1.000		1.000	
≥ 6 cm	2.112(1.374,3.246)	0.001	1.968(1.282,3.020)	0.002
				
Surgery type				
Amputation	1.000		1.000	
Limb sparing	0.439(0.292,0.660)	< 0.01	0.501(0.333,0.753)	0.001
				
Post-operative chemotherapy				
Not standard ^a^	1.000		1.000	
Standard ^b^	0.612(0.390,0.960)	0.033	0.646(0.412,1.013)	0.057
				
Pre-ALP				
Normal	1.000		1.000	
High	1.938(1.099,3.418)	0.022	1.985(1.126,3.500)	0.018
Very high	3.402(1.915,6.044)	< 0.01	3.267(1.840,5.800)	< 0.01
				
Lung metastasis				
No	1.000		1.000	
Yes	2.380(1.531,3.698)	< 0.01	5.562(3.422,9.040)	< 0.01
				
Local recurrence				
No	1.000		1.000	
Yes	1.255(0.652,2.418)	0.496	2.274(1.168,4.427)	0.016

OS overall survival, DFS disease-free survival, ALP alkaline phosphatase, *CI *confidence interval

^a ^post-operative chemotherapy cycles < 8

^b ^post-operative chemotherapy cycles ≥ 8

**Table 3:** Multivariate Cox regression analysis of potential prognostic factors for 177 osteosarcoma patients

Clinical characteristics	OS		DFS	
	
	Hazard ratio (95% *CI *)	*P*	Hazard ratio (95% *CI *)	*P*
Histological type				
Osteoblastic	3.615(1.232,10.603)	0.019	2.653(0.915,7.693)	0.072
Chondroblastic	2.262(0.673,7.602)	0.187	1.449(0.439,4.781)	0.543
Fibroblastic	1.670(0.425,6.564)	0.463	1.093(0.285,4.198)	0.897
Dilated blood vessels	3.506(0.932,13.195)	0.064	3.950(1.061,14.773)	0.041
Others	1.000		1.000	
				
Tumor size				
< 6 cm	1.000		----	----
≥ 6 cm	1.685(1.071,2.650)	0.024		
				
Surgery type				
Amputation	1.000		1.000	
Limb sparing	0.375(0.247,0.570)	< 0.01	0.392(0.257,0.597)	< 0.01
				
Pre-ALP				
Normal	1.000		1.000	
High	1.461(0.806,2.647)	0.212	1.542(0.847,2.806)	0.157
Very high	1.979(1.063,3.684)	0.031	2.090(1.135,3.848)	0.018
				
Lung metastasis				
No	1.000		1.000	
Yes	2.232(1.395,3.572)	0.001	5.456(3.266,9.114)	< 0.01

OS overall survival, DFS disease-free survival, ALP alkaline phosphatase, *CI *confidence interval

## Discussion

Osteosarcoma is characterized by the production of osteoid tissue or immature bone [19]. Biochemical markers which reflect global skeletal activity are sensitive indicators of early bone metabolism disturbances. Among them, products of the osteoblastic cells activity, serum alkaline phosphatase (ALP) which is one of the markers of bone formation is considered to be clinically useful [5]. Some authors had proved that serum ALP was raised in osteosarcoma patients and predicted a poorer survival [20,21]. Other authors have also reported the most important poor prognostic factor for survival was elevated ALP at diagnosis [5,22,23]. It appeared that ALP was a prognostic factor in multivariate analysis [24].

As known, serum levels of ALP are greater in infants and children than in adults [15]. Peak values occur at puberty, perhaps due to high skeletal growth velocity and rapid bone turnover during this period [25,26]. However, all the studies before [8,26,27] do not stratify for these differences, so the effect of age on ALP levels can not be evaluated. In our study, the age of patients ranged from 6 years to 56 years. To minimizing the influence of age on serum ALP levels, we defined upper normal limit of the serum ALP in patients younger than 18 years is 150 U/L and older than 18 years as 110 U/L. For the first time, we divided pre-ALP into three groups based on different normal upper limit. This method focused on the relationship between the quantity of the changes of ALP and the prognosis of the patients, not only on the limited level of ALP. This grouping can display the relationship between ALP and other clinical features much more correctly than the ALP (yes/no) grouping.

With effective chemotherapy or surgery for osteosarcoma patients, the decrease of the tumor cells in osteosarcoma patients caused the process of pathological osteogenesis slow down [28]. In our study, all the patients received neo-adjuvant chemotherapy, surgery and post-operative chemotherapy. We found that the patients whose elevated pre-ALP decreased after chemotherapy had significantly longer DFS and OS than the patients whose pre-ALP did not decrease following treatment. Decrease of serum ALP during clinical treatment may indicate low osteoblastic activity and insufficient mineralization, and also it may be a symptom of a positive reaction to treatment and disease remission. Thus, the change of serum ALP can be used as the marker for the response to clinical treatment in osteosarcoma patients. It should be helpful to evaluate the efficacy of different therapeutic protocols and plan new more aggressive therapeutic strategy. Bramer [18] has found similar results, but the limitation was that only patients over the age of 18 were studied, making their results only valid for adults, whereas the majority of osteosarcoma patients are under the age of 18. Moreover, they did not examine the predictive factors using multivariate Cox regression analysis.

Zhang, et al [29] and Hsieh, et al [11] have shown that released serum ALP was not only correlated with survival, but also with the recurrence and metastasis in osteosarcoma patients. Our study has pointed out the similar view on the correlationship between serum ALP and metastasis. While because there were so few patients with local recurrence in each pre-ALP group, we could not find the positive relationship between serum ALP and local recurrence. Moreover, we examined the MMP-9 expression levels in different pre-ALP groups. There was a significant correlation between the expression of MMP-9 and pre-ALP in these osteosarcoma samples. The positive rate and index of MMP-9 expression in osteosarcoma tissues were highest in very high pre-ALP group. The patients with high level of serum ALP at diagnosis had much higher risk of lung metastasis. Osteosarcoma metastasis is associated with the infiltration and expansion of tumor cells, which leading to the activation of osteoclasts. The activation of osteoclasts increased the severity of osteolysis accompanying with elevated ALP [30,31]. Meanwhile, the matrix metalloproteinases (MMPs) can be secreted by the cancer cell to dissolve extracellular matrix, which may have an interaction with elevated ALP. Therefore, to improve the DFS, pre-ALP would be helpful to identify patients with a poor prognosis who could be treated with more aggressive therapy.

Variation in methodology has led to inconsistent results and difficulty in interpreting the true prognostic effect of many of the variables evaluated [8,32]. Few studies have a look at whether serum ALP is equally pertinent in different therapeutic populations. In contrast, our study found it remained true that pre-ALP was strongly correlated with the survival of osteosarcoma patients even after stratifying the patients based upon surgery type, post-chemotherapy cycles, lung metastasis and local recurrence.

## Conclusions

In summary, the current study demonstrates the level of ALP is an independent prognostic factor for the survival of osteosarcoma patients, and is correlated with MMP-9 expression in osteosarcoma tissues. The ALP measured at diagnosis, or after clinical treatment and the change of ALP after treatment are possible valuable factors in predicting the clinical treatment response, lung metastasis and survival in osteosarcoma. Identifying ALP would be helpful in predicting the chance of metastasis and survival, especially early in treatment.

## Abbreviations

(ALP): alkaline phosphatase; (MMP-9): Matrix Metalloproteinase-9; (pre-ALP): pre-chemotherapy serum ALP; (DFS): disease-free survival; (OS): overall survival; (PBS): phosphate-buffered saline.

## Competing interests

The authors declare that they have no competing interests.

## Authors' contributions

TP and JS conceived the study. JH and CL performed the staining. BY and PT collected the cases and clinical information. JH interpreted the staining results and performed the statistical analysis. JH performed the literature review and wrote the manuscript. All authors read and approved the final manuscript.
